# Impact of the Timing of Enzyme Replacement Therapy Initiation and Cognitive Impairment Status on Outcomes for Patients with Mucopolysaccharidosis II (MPS II) in the United States: A Retrospective Chart Review

**DOI:** 10.36469/001c.36540

**Published:** 2022-08-29

**Authors:** Karen S. Yee, David Alexanderian, Yidie Feng, Xiaowei Ren, Bernd Schweikert, Olulade Ayodele

**Affiliations:** 1 Takeda Development Center Americas, Inc., Cambridge, Massachusetts; 2 Takeda Development Center Americas, Inc., Lexington, Massachusetts; 3 ICON plc, Vancouver, British Columbia, Canada

**Keywords:** cognitive impairment, disease burden, enzyme replacement therapy, Hunter syndrome, idursulfase, lysosomal storage disease, MPS II

## Abstract

**Background:** Mucopolysaccharidosis II (MPS II; Hunter syndrome; OMIM 309900) is a rare, X-linked, lysosomal storage disease caused by deficient iduronate-2-sulfatase activity. Accumulation of glycosaminoglycans results in multisystemic disease manifestations, which may include central nervous system involvement and cognitive impairment (CI). Patients with MPS II experience a high disease burden, leading to extensive healthcare resource utilization (HRU) and reduced quality of life.

**Objectives:** This study aimed to assess the impact of timing of enzyme replacement therapy (ERT) initiation and CI status on the clinical characteristics and HRU of patients with MPS II.

**Methods:** A retrospective medical chart review of 140 male patients who received a diagnosis of MPS II between 1997 and 2017 was performed at 19 US sites; data on disease manifestations and HRU stratified by age at ERT initiation or CI status were analyzed for the full study population and a subgroup of patients who received a diagnosis of MPS II before the age of 6 years.

**Results:** In patients initiating ERT before 3 years of age, there was a trend toward lower symptom burden and HRU compared with patients who initiated ERT at an older age. Evaluation of developmental and behavioral signs and symptoms in the full study population showed that communication delay (70.0% of patients), cognitive delay (62.1%), behavioral problems (52.9%), and toileting delay (50.0%) were particularly common; earliest documented signs and symptoms were motor delay (median [range] age at first documentation: 4.2 [0.9-18.7] years) and behavioral problems (4.4 [0.6-13.7] years). Patients with CI generally experienced greater symptom burden and higher HRU than those without CI, with the most notable differences documented for communication and toileting delays. Formal cognitive testing was documented in <30% of cognitively impaired patients diagnosed with MPS II before the age of 6 years.

**Conclusions:** Our findings reinforce previous recommendations for ERT to be initiated early to maximally benefit patients with MPS II, especially those younger than 3 years old. Cognitively impaired patients experience a particularly high disease burden and HRU. Patient care could be improved with early cognitive assessments and the development of treatments that address cognitive decline.

## BACKGROUND

Mucopolysaccharidosis II (MPS II; Hunter syndrome; OMIM 309900) is a rare, X-linked, lysosomal storage disease with an estimated birth prevalence of between 0.10 and 2.16 per 100 000 live births.^1^ Deficient activity of the lysosomal enzyme iduronate-2-sulfatase (I2S) in MPS II leads to glycosaminoglycan accumulation throughout the body.^2,3^ MPS II is a multisystemic and progressive disease; somatic signs and symptoms include skeletal deformities, joint stiffness, coarse facial features, hepatomegaly, splenomegaly, abdominal hernia, respiratory tract infections, obstructive airway disease, and cardiac disease.^2–4^ Severe speech and language delays, sleeping problems, and behavioral problems such as hyperactivity, impulsivity, and frustration have also been associated with MPS II.^2,5–11^

Two clinically distinct forms of MPS II have been described: a neuronopathic form, characterized by central nervous system involvement and cognitive impairment (CI), and a non-neuronopathic form.^12,13^ Patients with neuronopathic disease have similar somatic manifestations to those with the non-neuronopathic form, but the former experience a more severe natural disease progression and usually die in childhood, whereas those with the non-neuronopathic form typically survive into adulthood.^13–15^ More recently, the categorization of MPS II into two distinct forms is recognized to describe two extremes of a disease spectrum, with the signs and symptoms of MPS II now considered to reflect a continuum of disease severity.^16–19^ Regardless of MPS II presentation (neuronopathic or non-neuronopathic), patients experience a high disease burden, resulting in extensive healthcare resource utilization (HRU) and reduced quality of life.^20–23^

Treatment for MPS II is available in the form of enzyme replacement therapy (ERT) with recombinant human I2S (idursulfase; marketed as ELAPRASE®, Takeda Pharmaceuticals U.S.A., Inc., Lexington, Massachusetts).^24^ Idursulfase is administered weekly as an intravenous (IV) infusion and has been linked to increased survival and improvements in many somatic disease manifestations, including joint stiffness, liver and spleen enlargement, respiratory symptoms, and heart hypertrophy.^15,16,25–32^ Intravenous idursulfase is not expected to affect cognitive decline because it is unlikely to cross the blood-brain barrier in therapeutic quantities.^24,25^

The clinical needs and HRU of 140 patients with MPS II were previously evaluated in a retrospective chart review performed in the United States over a 20-year period (1997-2017).^23^ This study showed that both disease burden and HRU in these patients are high.^23^ Furthermore, the clinical burden and HRU were shown to be similar between patients who received ERT with IV idursulfase (n = 108) and those who had never received ERT (n = 32); however, this comparison was limited by the heterogeneity of the patient population.^23^

To inform healthcare decision-making, there is a need for greater understanding of the influence of timing of ERT initiation and CI status on the outcomes of patients with MPS II. Here, we report further analysis of the retrospective chart review to assess the impact of timing of ERT initiation (before the age of 3 years, between the ages of 3 and 6 years, or older than 6 years) and CI status on the clinical characteristics and HRU of patients with MPS II.

## METHODS

### Study Design and Population

A retrospective medical chart review of patients with MPS II was carried out at 19 sites across 14 states in the United States. Details of the study design and the study population have been previously described in full.^23^ Study sites were specialist clinics and were selected based on responses to a suitability questionnaire that determined the interests of the study investigator (someone who was a specialist in the treatment of patients with MPS II at any one of a number of major US-based medical sites), the number of eligible patients with MPS II, whether there was a suitable ethics approval process in place, and whether staff had capacity for the necessary data extraction. Eligible patients were identified by the participating physicians and site staff. Male patients of any age, with a diagnosis of MPS II (defined as a documented deficiency of I2S from a laboratory report and/or genetic analysis, phone report, or other communication) between 1997 and 2017 and who were patients at participating sites, were eligible for inclusion in the overall analysis population (n = 140). Due to the rarity of the disease, no other inclusion or exclusion criteria were implemented. Data from both living and deceased patients were included. To reduce heterogeneity within the patient population, some analyses (see below) were performed in a subgroup of patients who received a diagnosis of MPS II before the age of 6 years (n = 118).

### Ethics Approval and Consent to Participate

This study was approved by the Western Institutional Review Board, as well as by all relevant local institutional review boards (IRBs). Waivers of consent for the data collection were granted by all IRBs.

### Data Collection and Analyses

The data collection process was previously described in detail.^23^ Relevant information from the charts of all included patients was compiled using an online database. Data were collected from as early as available in patients’ charts until the last date of data entry, loss to follow-up, or death, whichever occurred first. Data were entered into an electronic case report form (DataTrak International, Mayfield Heights, Ohio) and cleaned by the authors via automated queries, manual review, and reclassification. If potential data entry errors in the electronic case report form were detected, or clarifications were required, queries were posed to the relevant site through the online system. Sites then made corrections to the entered data as necessary. Where appropriate, free-text entries for clinical characteristics, outcomes, or resource use that had been listed as “other” were reclassified into existing categories. New categories were created for responses that were mentioned frequently in free-text responses. All entries that were not recategorized remained listed as “other.”

Analyses of data from patients’ charts were performed using SAS version 9.4 (SAS Institute Inc, Cary, North Carolina). Patients were classified as having CI if they had cognitive delay documented at any point during the study, regardless of whether they had undergone formal testing.

For the full study population, age at first documentation of symptoms was analyzed for all ERT-treated patients (defined as having ≥1 treatment with idursulfase documented in their chart) and patients were stratified by age at ERT initiation into the following categories: <3 years, 3-6 years and >6 years. These age categories were selected to align with previous analyses,^33^ predefined clinical study subgroups,^34,35^ and chronological age ranges of cognitive assessment instruments that are commonly used in the United States.^36–38^

Disease manifestations, proportion of patients with specific HRU, and the annual frequency of HRU were also analyzed and stratified by CI status (diagnosed; never diagnosed). Patients were excluded from the HRU analyses if they had only 1 documented visit or if data were collected at a site where more than 25% of HRU was categorized as unknown for one of the core variables (surgery, hospitalization, and outpatient visits). For the frequency of resource use analysis, 1 additional patient was excluded because all of their HRU was classified as unknown. The annual frequency of HRU was calculated by dividing the number of cases of resource use by the number of follow-up years (between the first and last documented visit).

For the subgroup of patients who received a diagnosis of MPS II before the age of 6 years, disease manifestations and HRU were analyzed in all ERT-treated patients stratified by age at ERT initiation. The same criteria described for the full study population were also used to exclude patients from HRU analyses in this subgroup. Disease manifestations and HRU stratified by CI status (diagnosed; never diagnosed) were also analyzed. Formal cognitive and/or communication assessments were analyzed for all patients who had these tests documented in their chart; types of tests were analyzed for patients with CI who had at least 1 assessment type recorded.

## RESULTS

A summary of the patient groups in this study is shown in **Supplementary Table S1**.

### Full Study Population

**Patient characteristics:** Patient characteristics for the full study population (n = 140) overall and stratified by CI status (diagnosed; never diagnosed) are summarized in Table 1. Of the patients in the study population, 87 of 140 (62.1%) had CI recorded in their patient chart and 53 of 140 (37.9%) had no CI recorded. Of the patients with diagnosed CI, 48 of 87 (55.2%) had CI first documented before 6 years of age and 33 of 87 (37.9%) had CI first documented at 6-11 years of age. Patients with CI received an MPS II diagnosis at a younger mean age (SD) compared with those without CI (3.0 [2.1] and 5.2 [8.9] years, respectively), although the median age at diagnosis was similar between these groups. Of the patients with diagnosed CI in this study, 14 of 87 (16.1%) received a diagnosis of CI before their MPS II diagnosis, 71 of 87 (81.6%) received a diagnosis of CI after their MPS II diagnosis, and 2 of 87 (2.3%) had no MPS II diagnosis date available. The proportion of patients who had received ERT was similar for those with CI (69/87 [79.3%]) and for those without CI (39/52 [75.0%]).

**Table 1.** Summary of Patient Characteristics for the Full Study Population

	**All Patients (n = 140)**	**CI Diagnosed^a^ (n = 87)**	**CI Never Diagnosed^a^ (n = 53)**
Alive, n (%)^b^	122 (87.1)	76 (87.4)	46 (86.8)
Mean (SD)	11.5 (7.7)	10.9 (5.1)	12.3 (10.8)
Median (range)	9.8 (0.6, 62.5)	10.2 (2.3, 22.8)	8.6 (0.6, 62.5)
Deceased, n (%)	18 (12.9)	11 (12.6)	7 (13.2)
Last documented ages, years (deceased)			
Mean (SD)	12.5 (4.9)	12.6 (4.1)	12.2 (6.3)
Median (range)	13.4 (3.3, 20.2)	12.9 (3.3, 18.6)	14.2 (4.6, 20.2)
Cause of death, n (%)			
Related to MPS II	7 (38.9)	4 (36.4)	3 (42.9)
Unclear if related to MPS II	5 (27.8)	2 (18.2)	3 (42.9)
Unknown	6 (33.3)	5 (45.5)	1 (14.3)
Age at diagnosis, years^c^	n = 136	n = 85	n = 51
Mean (SD)	3.8 (5.8)	3 (2.1)	5.2 (8.9)
Median (range)^d^	2.8 (−0.1, 59.7)	2.8 (0.0, 12.8)	3.2 (−0.1, 59.7)
Family history of MPS II, n (%)^e^	53 (37.9)	33 (37.9)	20 (37.7)
Sibling(s) with MPS II	33 (62.3)	20 (60.6)	13 (65.0)
Uncle(s) with MPS II	15 (28.3)	10 (30.3)	5 (25.0)
Ethnicity, n (%)			
White/non-Hispanic	76 (54.3)	40 (46.0)	36 (67.9)
Black/African American	25 (17.9)	20 (23.0)	5 (9.4)
Hispanic/Latino	21 (15.0)	15 (17.2)	6 (11.3)
Mixed	7 (5.0)	6 (6.9)	1 (1.9)
Asian	5 (3.6)	3 (3.4)	2 (3.8)
Other	5 (3.6)	2 (2.3)	3 (5.7)
Unknown	1 (0.7)	1 (1.1)	0 (0.0)
Insurance status, n (%)			
Private	58 (41.4)	32 (36.8)	26 (49.1)
Medicaid	57 (40.7)	39 (44.8)	18 (34.0)
None	1 (0.7)	0 (0)	1 (1.9)
Other	9 (6.4)	6 (6.9)	3 (5.7)
Multiple insurances	13 (9.3)	9 (10.3)	4 (7.5)
Unknown	2 (1.4)	1 (1.1)	1 (1.9)
US region			
East	26 (18.6)	21 (24.1)	5 (9.4)
South	32 (22.9)	16 (18.4)	16 (30.2)
Midwest	52 (37.1)	30 (34.5)	22 (41.5)
Southwest	17 (12.1)	12 (13.8)	5 (9.4)
West	13 (9.3)	8 (9.2)	5 (9.4)
Patients receiving ERT^f^	n = 139	n = 87	n = 52
Number (%)	108 (77.7)	69 (79.3)	39 (75.0)

**Figure 1.**
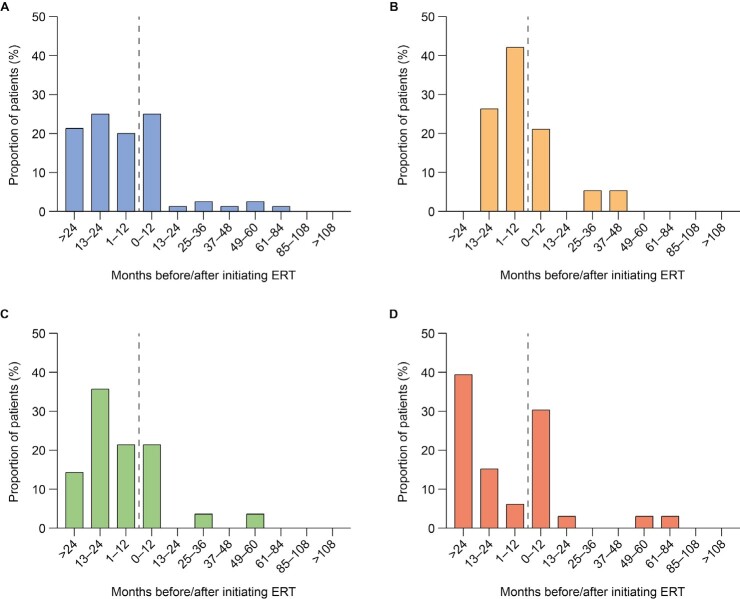
First Documentation of Organ Dysfunction Relative to Time of ERT Initiation Results are presented for (**A**) all patients (n = 80), (**B**) patients starting ERT aged <3 years (n = 19), (**C**) patients starting ERT aged 3-6 years (n = 28), and (**D**) patients starting ERT aged >6 years (n = 33). No symptoms of organ dysfunction were documented for 28 patients. Proportion of patients who exhibited symptoms is shown. Dashed line indicates the point at which ERT was initiated. Abbreviation: ERT, enzyme replacement therapy.

**First documentation of disease manifestations relative to time of ERT initiation:** Time between ERT initiation and first documentation of symptoms was analyzed for all ERT-treated patients (n = 108) and stratified by age at ERT initiation (examples for organ dysfunction shown in Figure 1A-D; examples for musculoskeletal symptoms, cardiovascular symptoms, ear, nose, and throat [ENT] symptoms, infections and CI are shown in **Supplementary Figures S1-S5**). First documentations of disease manifestations (including musculoskeletal symptoms, ENT symptoms, infections, and organ dysfunction) were generally more common before initiation of ERT than after initiation, regardless of the age at ERT start. However, there were some exceptions. The first documentation of cardiovascular symptoms was generally less common before initiation of ERT than after in all patients and in patients starting ERT before 3 years of age. Similarly, first documentation of infections was generally less frequent before initiation of ERT than after in patients starting ERT before 3 years of age. Documentation of CI in all patients and all age groups was also generally less common before than after initiation of ERT. For all somatic disease manifestations and CI, there appeared to be a higher proportion of patients initiating ERT after 6 years of age experiencing new, documented disease manifestations in the 12 months from ERT initiation compared with the 12 months before ERT initiation (example for organ dysfunction shown in Figure 1D).

**Figure 2.**
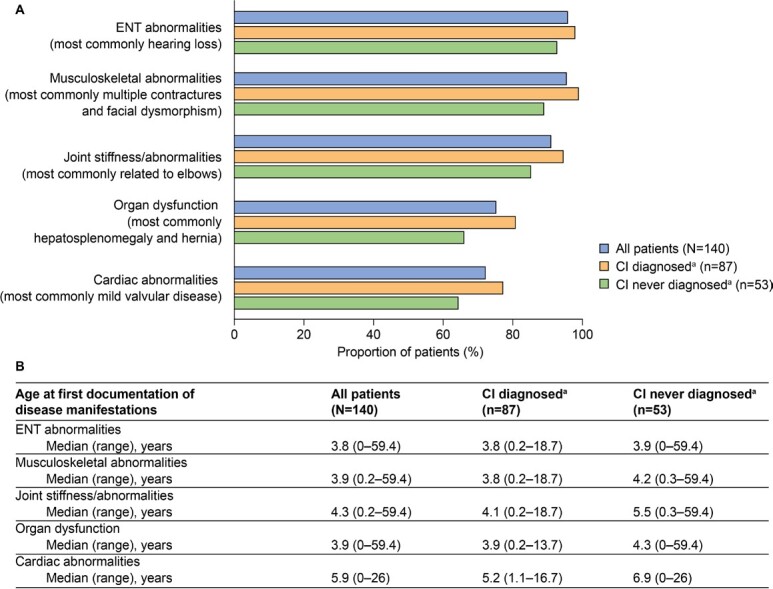
Common Somatic Disease Manifestations and Age at First Documentation by CI Status Results are presented for (**A**) common somatic disease manifestations and (**B**) age at first documentation of disease manifestations. Abbreviations: CI, cognitive impairment; ENT, ear, nose, and throat. ^a^CI never diagnosed/diagnosed: “cognitive delay” documented never/at least once in patient’s chart.

**Common somatic disease manifestations and age at first documentation by CI status:** The estimated proportion of somatic disease manifestations and age at first documentation in the full study population (n = 140) were previously analyzed^23^; these data for common somatic disease manifestations are also presented in Figure 2. Further analysis of these data revealed a trend toward a higher prevalence of musculoskeletal abnormalities, joint stiffness/abnormalities, decreased respiratory function, cardiovascular abnormalities, ENT abnormalities, and organ dysfunction (differences ranged between 5.2% and 14.6%) in patients with CI than in those without CI (Figure 2A). These disease manifestations were also generally documented at a younger median age in patients with CI than in those without CI (Figure 2B, differences ranged from 0.1 to 1.7 years). Notably, the median age at first documentation appeared to be lower by 4.4 years or more in patients with CI than in those without CI for tracheomalacia, left ventricular hypertrophy, carpal tunnel syndrome, and renal dysfunction. A notable exception to this trend was ventricular dilation, for which the median age at first documentation appeared to be lower by 5.0 years in patients without CI than in those with CI.

**Figure 3.**
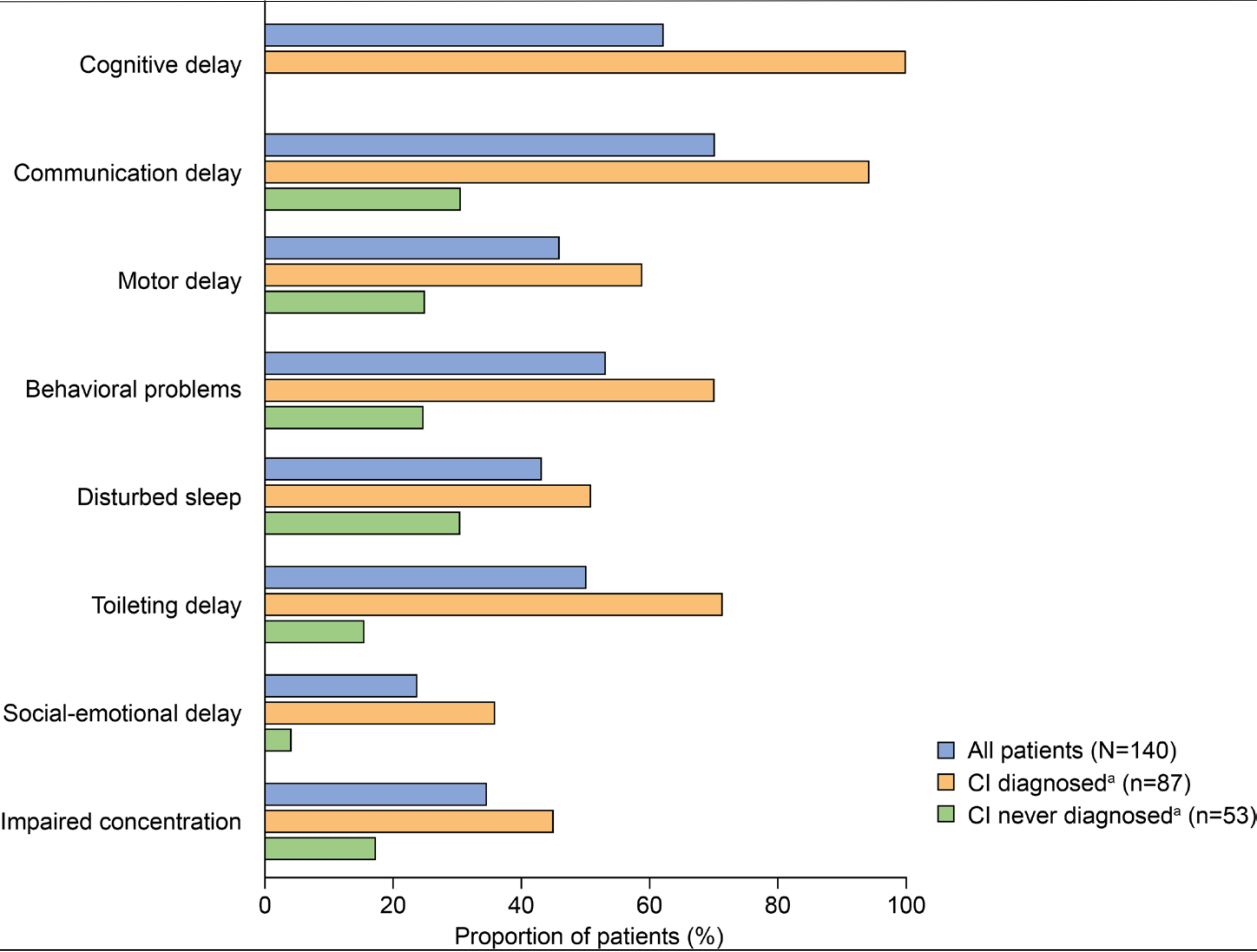
Developmental and Behavioral Signs and Symptoms by CI Status Abbreviation: CI, cognitive impairment. ^a^CI never diagnosed/diagnosed: “cognitive delay” documented never/at least once in patient’s chart.

Developmental and behavioral signs and symptoms documented in patients with MPS II (all patients and stratified by CI status) are summarized in Figure 3. The prevalence of communication delay (70%), cognitive delay (62.1%), behavioral problems (52.9%), and toileting delay (50%) in the full study population was particularly high. Motor delay, behavioral problems, disturbed sleep, social-emotional delay, and impaired concentration were also documented in more than 20% of all patients.

Most developmental and behavioral signs and symptoms were first documented at a median age of between 2.2 and 5.3 years. An exception to this was seizures, which were first documented at a median (range) age of 10.4 (1.8-20.9) years. Excluding communication delay (for which data were available for only 1 patient), the earliest documented developmental/behavioral signs or symptoms were motor delay (median [range] age at first documentation: 4.2 [0.9-18.7] years) and behavioral problems (4.4 [0.6-13.7] years).

Similar to somatic disease manifestations, developmental and behavioral signs and symptoms were documented regardless of CI status but were generally more common in patients with CI (Figure 3). The most notable differences were documented for communication and toileting delays, which appeared to be over 50% more prevalent in patients with CI than in those without CI.

**Figure 4.**
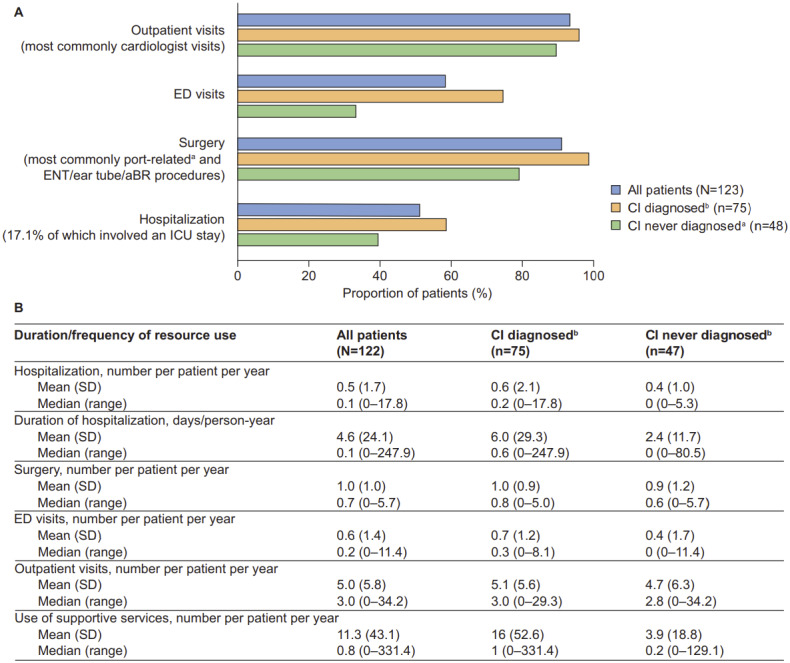
HRU and Duration/Frequency of Resource Use by CI Status Results are presented for (**A**) HRU and (**B**) duration/frequency of resource use. Abbreviations: aBR, auditory brain stem response; CI, cognitive impairment; ED, emergency department; ENT, ear, nose, and throat; HRU, healthcare resource utilization; ICU, intensive care unit. ^a^All patients who underwent surgeries for port-related issues also underwent surgery at least once for other issues. ^b^CI never diagnosed/diagnosed: “cognitive delay” documented never/at least once in patient’s chart.

**Healthcare resource utilization by CI status:** Overall HRU and annual frequency of resource use for the full study population have been previously described in detail and are summarized in Figure 4.^23^ Both were also evaluated for patients with and without CI (n = 123 and n = 122, respectively). The findings revealed a trend toward patients with CI being more likely to have used healthcare resources, including outpatient visits, emergency department (ED) visits, surgery, and hospitalization, than those without CI (Figure 4A). The largest difference was documented for ED visits, which were accessed by 74.7% of patients with CI and 33.3% of patients without CI. Cardiologist visits were the most common type of outpatient visit (accessed by 84.0% of patients with CI and 58.3% of patients without CI). Patients with CI also appeared to have a longer mean (SD) duration of hospitalization than patients without CI (6.0 [29.3] vs 2.4 [11.7] days/person-year, respectively) and greater mean use of supportive services (16 [52.6] vs 3.9 [18.8] per person per year, respectively (Figure 4B). The 3 most accessed supportive services among those with CI were occupational therapy (70.7%, n = 53), speech and hearing therapy (70.7%, n = 53), and special education (30.7%, n = 23).

### Subgroup Population: Patients Who Received a Diagnosis of MPS II Before the Age of 6 Years

To investigate the possible influence of early diagnosis, we also analyzed the effect of age at ERT initiation on disease manifestations and HRU in a subgroup of patients who received a diagnosis of MPS II before the age of 6 years (n = 118).

**Patient characteristics:** In total, 29 of 90 treated patients initiated ERT before 3 years of age, 34 at 3-6 years of age, and 27 after 6 years of age (**Supplementary Table S1**). At the time of the most recent data entry, 83 patients were alive (mean age [SD]: 9.9 [5.2] years) and 7 were deceased (mean age [SD]: 14.7 [4.1] years). The mean age (SD) at ERT initiation was 61.2 (43.2) months. Mean (SD) time from diagnosis to ERT initiation was lower for patients who received a diagnosis of MPS II after approval of idursulfase in July 2006 than patients who received a diagnosis before idursulfase approval (9.7 [7.3] vs 12 [0.0], 17.2 [12.3] vs 31.6 [13.7], and 64.3 [26.4] vs 73.5 [47.1] months for patients who started ERT at <3, 3-6, and >6 years of age, respectively). Follow-up times from first documentation of ERT varied for patients who initiated ERT at different ages (median of 36.8, 52.8, and 69.3 months for patients who started ERT at <3, 3-6, and >6 years of age, respectively). Cognitive impairment was recorded for a slightly higher proportion of patients in the subgroup population of patients with an MPS II diagnosis before the age of 6 years (n = 118) than in the full study population (79.7% vs 62.1%, respectively).^23^

**Figure 5.**
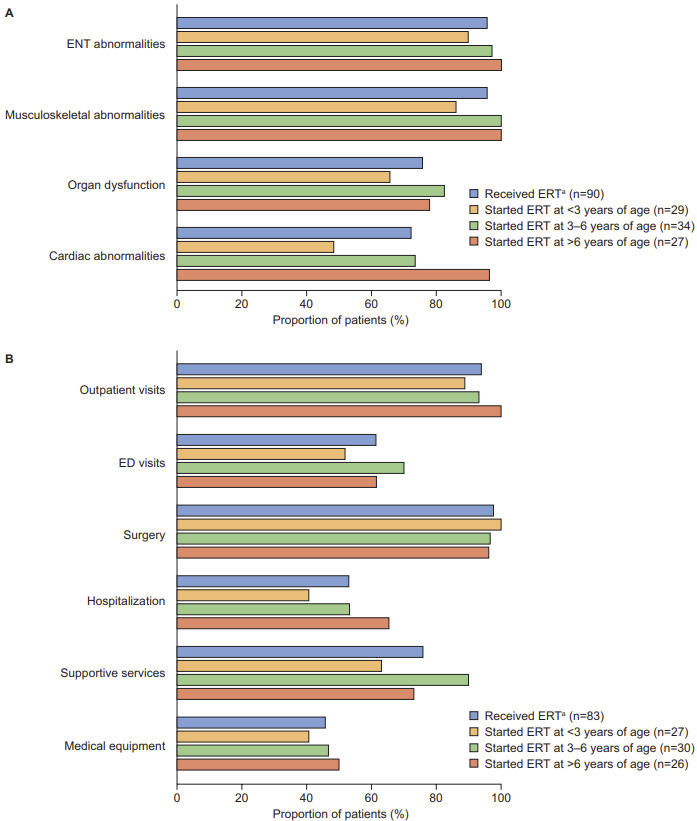
Prevalence of Common Disease Manifestations and HRU in Patients Who Received a Diagnosis of MPS II Before the Age of 6 Years (**A**) Common disease manifestations and (**B**) HRU are presented for the subgroup of patients who received a diagnosis of MPS II before 6 years of age and stratified by age at ERT initiation. Abbreviations: ENT, ear, nose and throat; ED, emergency department; ERT, enzyme replacement therapy; HRU, healthcare resource utilization; MPS II, mucopolysaccharidosis II. ^a^Received ERT: at least 1 treatment with idursulfase documented in patient’s chart.

**Disease manifestations by age at ERT initiation:** To explore the potential effect of age at ERT initiation on disease manifestations, the prevalence of common disease manifestations and the age at which they were first documented was analyzed for patients who received a diagnosis of MPS II before the age of 6 years and who initiated ERT at <3, 3-6, and >6 years of age. Somatic disease manifestations (ENT abnormalities, musculoskeletal abnormalities, organ dysfunction, and cardiac abnormalities) were generally documented in a lower proportion of patients who initiated ERT at before 3 years of age than in those who initiated ERT at an older age (Figure 5A). This effect was particularly notable for cardiac manifestations, which were documented in 48.3%, 73.5%, and 96.3% of patients who initiated ERT at <3, 3-6, and >6 years of age, respectively.

Somatic disease manifestations also tended to be documented earlier in patients who started ERT at a younger vs older age, with the median age at first documentation of symptoms approximately 2 years earlier in patients initiating ERT before 3 years of age than in those initiating ERT at 3-6 years of age, and approximately 2.5 years earlier in patients initiating ERT at 3-6 years of age than in those initiating ERT after 6 years of age.

**Healthcare resource utilization by age at ERT initiation:** Except for surgery, access to HRU (outpatient visits, ER visits, hospitalization, supportive services, and medical equipment) tended to be lower in patients who initiated ERT before 3 years of age than in those who initiated ERT at an older age (Figure 5B). This effect was particularly apparent for hospitalization (accessed by 40.7%, 53.3%, and 65.4% of patients who initiated ERT at <3, 3-6, and >6 years of age, respectively) and supportive services (accessed by 63%, 90%, and 73.1% of patients who initiated ERT at <3, 3-6, and >6 years of age, respectively).

**Cognitive impairment:** Similar to the full study population, 66.9% of patients who received a diagnosis of MPS II before the age of 6 years (n = 79) had CI, and 79.7% of these received ERT (n = 63). The majority of patients with CI had social-emotional delays, behavioral problems, and/or impaired concentration documented in their charts, with aggression (46.8% of patients) and hyperactivity (34.2% of patients) being particularly common. Aggression and hyperactivity were first documented at a median age (range) of 4.0 (2.3-9.0) and 4.4 (1.6-7.8) years, respectively, and the median age (range) at first documentation of all social-emotional delays, behavioral problems and/or impaired concentration was 4.0 (1.2-13.7) years. The prevalence of disease manifestations and HRU in patients with CI and in those without CI was similar in the subgroup of patients who received a diagnosis of MPS II before the age of 6 years and in the full study population.

Of the 118 patients who received a diagnosis of MPS II before the age of 6 years, only 26.5% underwent formal cognitive and/or communication assessments, with a median (range) age at first assessment of 4.3 (1.2-14.9) years. Cognitive and/or communication assessments were documented for 21 patients and, of the 14 different cognitive tools used to assess these patients, the most common were the Mullen Scales of Early Learning (used for 6.3% of patients) and the Wechsler Preschool and Primary Scale of Intelligence/Vineland Adaptive Behavior Scales (used for 5.1% of patients).

## DISCUSSION

Our findings demonstrate a trend for lower symptom burden and HRU (absolute and annual frequency) in patients initiating ERT before 3 years of age than in patients who initiated ERT at an older age. Furthermore, we demonstrated a trend for higher symptom burden and HRU in patients with CI than in those without CI. These findings extend those previously published from this chart review study, which showed that patients with MPS II in the United States experience a substantial clinical burden and high HRU.^23^

Patients with CI were diagnosed with MPS II at a younger mean age than those without CI. This is consistent with the early onset and/or diagnosis of MPS II in patients with severe, progressive disease, is well documented in the literature,^4,8,13,19^ and supports the use of age at onset or diagnosis as a predictor of the severity of the natural course of the disease. However, initiation of ERT soon after an early diagnosis may alter this disease course. In the full study population, early ERT initiation (before the age of 3 years) was generally associated with later documentation of somatic disease manifestations compared with ERT initiation after the age of 3 years. In contrast, analysis of patients who received a diagnosis of MPS II before 6 years of age showed that disease manifestations were documented earlier in patients who started ERT at a younger vs older age. The latter finding may be explained by a higher level of disease awareness for patients who received a diagnosis of MPS II before the age of 6 years, which could increase the documentation of somatic disease manifestations. It is also worth noting that the age at first documentation of disease manifestations in patient charts may not reflect the true age at onset.

In patients who started ERT before the age of 3 years, a lower prevalence of somatic disease manifestations and HRU was generally documented compared with patients who started ERT at an older age. Although previous findings suggested that clinical burden and HRU were similar in patients who received ERT and in those who did not, these findings were limited by the heterogeneity of the sample population.^23^ To investigate the possible effect of age at ERT initiation, we used a less heterogenous sample population (patients who received an MPS II diagnosis before 6 years of age). The proportion of patients with CI was comparable between the full study population (62%) and the subgroup population (67%). Our findings support recommendations for ERT to be administered as early as possible in the disease course to maximize halting or slowing down the progression of MPS II disease manifestations.^39^ However, these results should be interpreted with caution, owing to the variation in follow-up times from first documentation of ERT for patients who initiated ERT at different ages, which may allow more or less time for disease progression and HRU.

Patients with CI generally exhibited a higher clinical burden than those without CI, with somatic disease manifestations appearing at a younger age. HRU, including the use of supportive services, was also higher for patients with CI than for those without CI. The trend toward a higher prevalence of some somatic manifestations in patients with CI compared with patients without CI, in addition to the presence of the neurological symptoms, may have contributed to their higher HRU. However, it should be noted that other reports in the literature describe similar somatic symptoms in patients with and without neurocognitive impairment.^9,40–42^ Regardless of the symptom profile, frequent use of supportive services places a significant burden on caregivers, who are often responsible for transporting and/or accompanying patients to these sessions. Potential socioeconomic and racial/ethnic disparities in the burden of disease and access to resources—and therefore HRU—merit consideration. Over 50% of the patients in this study had private insurance or multiple insurance coverages and more than half were White/non-Hispanic. The need for increased awareness of, and improvements in, the disparities in care and access to resources in rare diseases is receiving increasing attention^43–45^; the impact of these factors on the management of MPS II warrants further investigation.

Developmental and behavioral signs and symptoms were generally more frequent in patients with CI than in those without; however, many patients without CI also experienced developmental delays, suggesting that these signs and symptoms contribute substantially to the disease burden in all patients with MPS II.

The study by Young et al^13^ in 1982 originally stated that approximately two-thirds of patients with MPS II have neuronopathic disease and present with central nervous system involvement and CI. In a later (2002-2010) study by Holt et al^8^ of 49 patients with MPS II, 37 patients (76%) were shown to have neurocognitive decline. The finding that 87 of 140 patients (62%) in the present study had CI could therefore be considered to be slightly lower than expected. This may reflect a lack of cognitive testing as well as a lack of recording of the results in the charts. Indeed, only approximately 25% of patients who received a diagnosis of MPS II before 6 years of age underwent a formal cognitive or communication test, indicating that these tests are not generally included in the routine care of MPS. Such low uptake of these tests could limit the ability to describe CI at the population level, including how CI affects disease severity and HRU. Encouraging the widespread use of these tests in clinical practice would enhance disease monitoring and the identification of specific healthcare resource needs, helping to improve the overall standard of care for patients with neuronopathic MPS II.

Predicting the development of CI in patients with MPS II is challenging, and further work is required to establish which of the developmental and/or behavioral characteristics described in this study might precede CI and would be useful for this purpose. Furthermore, to reduce the burden of disease for patients with CI, there is an unmet clinical need for new treatments that cross the blood-brain barrier. Treatments in development for neuronopathic MPS II include intracerebroventricular or intrathecal ERT, fusion proteins that facilitate blood-brain barrier penetration, and gene therapy.^46–50^

### Limitations

The key strengths of this study included the relatively large patient numbers for a rare disease, the 20-year period of study, and the long duration of patient follow-up.^23^ However, owing to the real-world nature of this study, the findings were limited by the variability of data recorded in patient charts and the differing documentation of HRU across sites. A possible lack of CI status in the charts for some patients, assessment of CI using multiple methods, both formal and nonformal, and analysis of only those with a formal cognitive test documented in their chart also limited the interpretation of these results. The potential impact of patient participation in ongoing clinical trials on the availability of patient data and types of cognitive tools used should also be acknowledged. Additional potential confounding factors include the variation in duration of ERT, differences in baseline characteristics between subgroups, and changes in the standards of clinical care since the approval of idursulfase in 2006. Thereafter, a shift in treatment occurred, from palliative care focused on alleviating the diverse clinical symptoms to the use of a disease-specific treatment with the potential to target the underlying cause and slow or prevent the progressive tissue and organ damage.^4^

## CONCLUSION

Our analysis of US patient data reveals a trend toward lower symptom burden and HRU in patients initiating ERT when they were younger than 3 years of age than in those initiating ERT at an older age. Although potential effects of differences in baseline characteristics between subgroups should be acknowledged, this finding adds to the available body of evidence supporting early ERT. Furthermore, our data extend our understanding of the disease manifestations and clinical needs of cognitively impaired patients with MPS II in the United States and highlight the unmet clinical need for increased cognitive testing and for treatments that prevent or slow cognitive decline.

### Author Contributions

K.S.Y. contributed to study conception and design. D.A. contributed to study conception and design. Y.F. performed the chart review analysis. X.R. contributed to study conception and design. B.S. contributed to study design and performed the chart review analysis. O.A. contributed to study conception and design. All authors contributed to interpretation of data, drafted the initial manuscript and revised it critically for important intellectual content, approved the final manuscript as submitted, and agree to be accountable for all aspects of the work.

### Disclosures

K.S.Y. is a full-time employee of Takeda Development Center Americas, Inc. and stockholder of Takeda Pharmaceuticals Company Limited. D.A. was a full-time employee of Takeda Development Center Americas, Inc. at the time of this analysis, and is a current employee of Affinia Therapeutics Inc. and a stockholder of Takeda Pharmaceuticals Company Limited. Y.F. was a full-time employee of ICON plc, a contract research organization contracted for this chart review, at the time of this analysis and is a current employee of IQVIA. X.R. is a full-time employee of Takeda Development Center Americas, Inc. and a stockholder of Takeda Pharmaceuticals Company Limited. B.S. is a full-time employee of ICON plc, a contract research organization contracted for this chart review. O.A. is a full-time employee of Takeda Development Center Americas, Inc. and a stockholder of Takeda Pharmaceuticals Company Limited.

### Availability of Data and Materials

The datasets, including redacted study protocol, redacted statistical analysis plan, and individual participants’ data supporting the results reported in this article will be made available within 3 months from initial request to researchers who provide a methodologically sound proposal. The data will be provided after its de-identification in compliance with applicable privacy laws, data protection, and requirements for consent and anonymization.

## Supplementary Material

Online Supplementary Material
