# Supplementary material for: Impact of the Timing of Enzyme Replacement Therapy Initiation and Cognitive Impairment Status on Outcomes for Patients with Mucopolysaccharidosis II (MPS II) in the United States: A Retrospective Chart Review
Source: J Health Econ Outcomes Res. 2022 Aug 29;9(2):67–76. doi: 10.36469/001c.36540 (PMC9424538; doi:10.36469/001c.36540)
Supplement: Online Supplementary Material [file jheor_2022_9_2_36540_98265.pdf]

Journal of Health Economics and Outcomes Research

Online Supplementary Material

Impact of the Timing of Enzyme Replacement Therapy Initiation and Cognitive Impairment Status on Outcomes for Patients With Mucopolysaccharidosis II (MPS II) in the United States: A Retrospective Chart Review. *JHEOR*. 2022;9(2):67-76. doi:10.36469/jheor.2022.36540

Table S1: Summary of Patient Groups

Figure S1: First Documentation of Musculoskeletal Symptoms Relative to Time of ERT Initiation

Figure S2: First Documentation of Cardiovascular Symptoms Relative to Time of ERT Initiation

Figure S3: First Documentation of ENT Symptoms Relative to Time of ERT Initiation

Figure S4: First Documentation of Infections Relative to Time of ERT Initiation

Figure S5: First Documentation of CI Relative to Time of ERT Initiation

This supplementary material has been provided by the authors to give readers additional information about their work.

This is an open-access article distributed under the terms of the Creative Commons Attribution 4.0 International License (CCBY-4.0). View this license's legal deed at http://creativecommons.org/licenses/by/4.0 and legal code at http://creativecommons.org/licenses/by/4.0/legalcode for more information.

Table S1. Summary of Patient Groups

Population	Additional Description	No. of Patients
**Full study population**	Male patients who received a diagnosis of MPS II, 1997-2017	140
Year of diagnosis		
1997-2001	—	17
2002-2006	—	36
2007-2011	—	48
2012-2016	—	34
2017	—	1
Missing	—	4
Year of birth		
Pre-1987	—	1
1987-1991	—	4
1992-1996	—	11
1997-2001	—	31
2002-2006	—	32
2007-2011	—	45
2012-2016	—	16
ERT-treated patients	≥1 documented treatment with idursulfase	108
Started ERT aged <3 years	—	30
Started ERT aged 3-6 years	—	34
Started ERT aged >6 years	—	44
Patients with CI	Cognitive delay documented at any point during the study	87
Patients without CI	Cognitive delay not documented at any point during the study	53
**Subgroup population**	Patients who received a diagnosis of MPS II at age <6 years	118
ERT-treated patients	≥1 documented treatment with idursulfase	90
Started ERT aged <3 years	—	29
Started ERT aged 3-6 years	—	34
Started ERT aged >6 years	—	27
Patients with CI	Cognitive delay documented at any point during the study	79
Patients without CI	Cognitive delay not documented at any point during the study	39

Abbreviations: CI, cognitive impairment; ERT, enzyme replacement therapy; MPS II, mucopolysaccharidosis II.

Figure S1. First Documentation of Musculoskeletal Symptoms Relative to Time of ERT Initiation

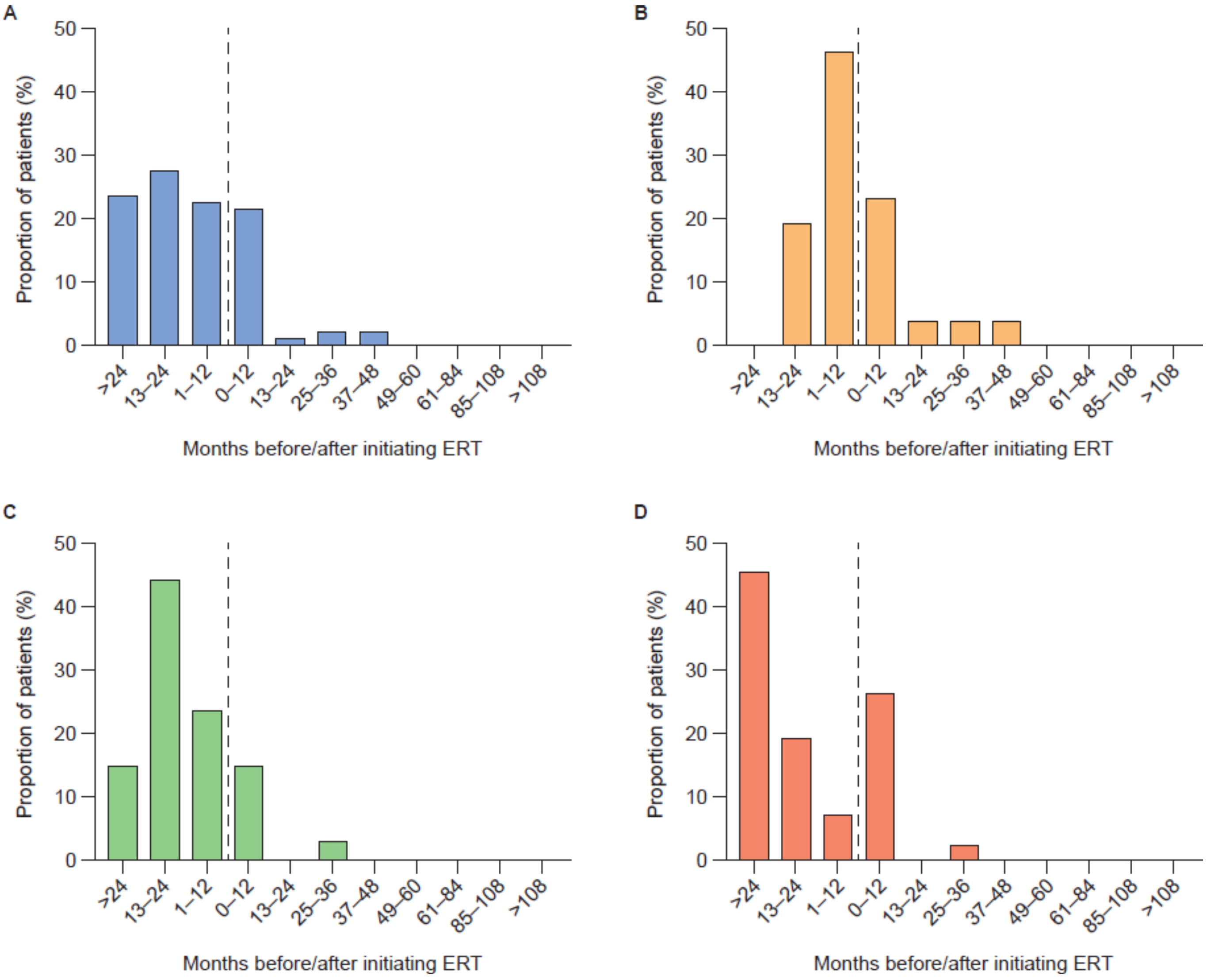

Results are presented for (**A**) all patients (n = 102), (**B**) patients starting ERT aged <3 years (n = 26), (**C**) patients starting ERT aged 3-6 years (n = 34), and (**D**) patients starting ERT aged >6 years (n = 42). No musculoskeletal symptoms were documented for 6 patients.

Proportion of patients who exhibited symptoms is shown. Dashed line indicates the point at which ERT was initiated.

Abbreviation: ERT, enzyme replacement therapy.

Figure S2. First Documentation of Cardiovascular Symptoms Relative to Time of ERT Initiation

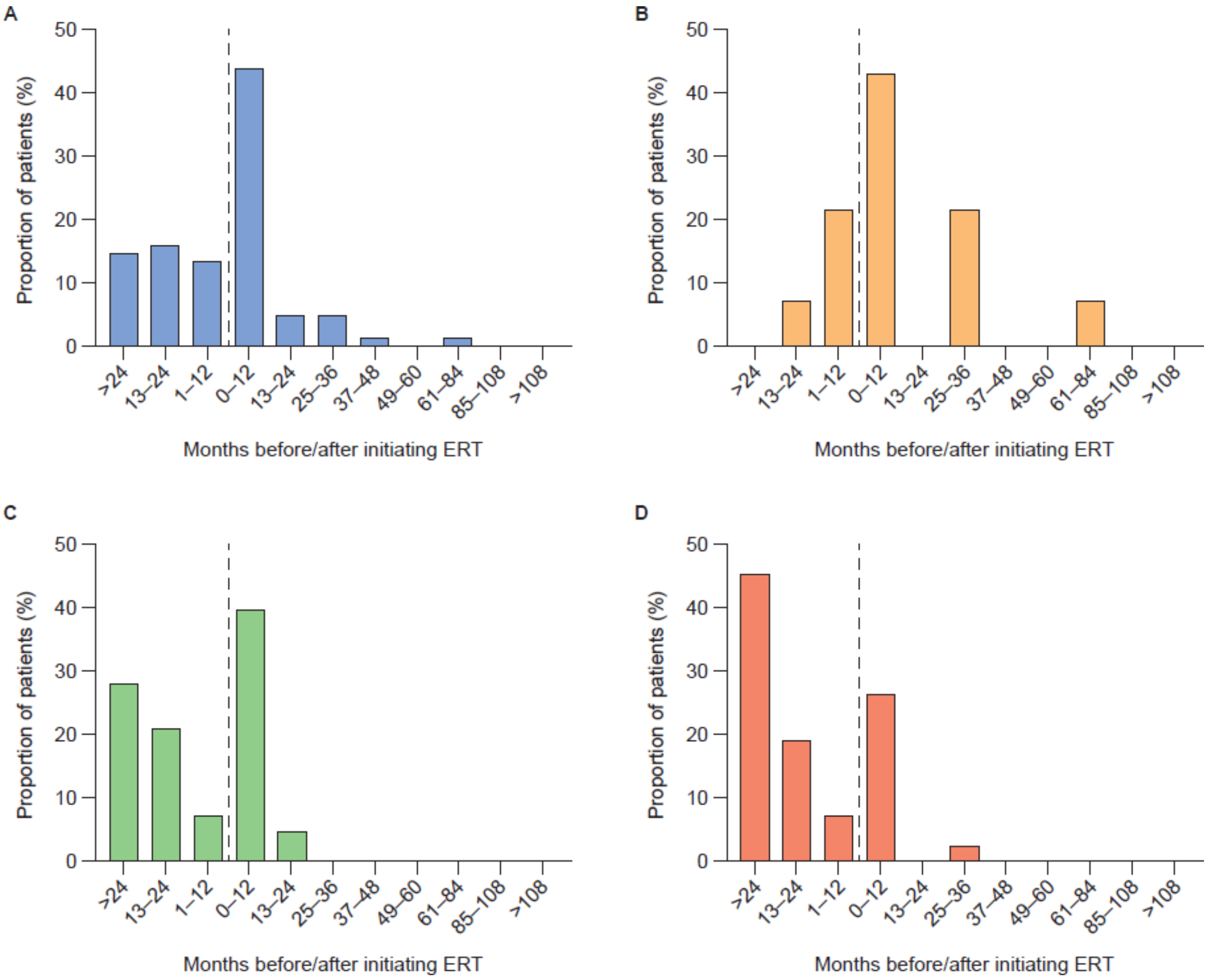

Results are presented for (A) all patients (n=82), (B) patients starting ERT aged <3 years (n=14), (C) patients starting ERT aged 3-6 years (n= 34), and (D) patients starting ERT aged >6 years (n = 42). No cardiovascular symptoms were documented for 26 patients.
Proportion of patients who exhibited symptoms is shown. Dashed line indicates the point at which ERT was initiated.
Abbreviation: ERT, enzyme replacement therapy.

Figure S3. First Documentation of ENT Symptoms Relative to Time of ERT Initiation

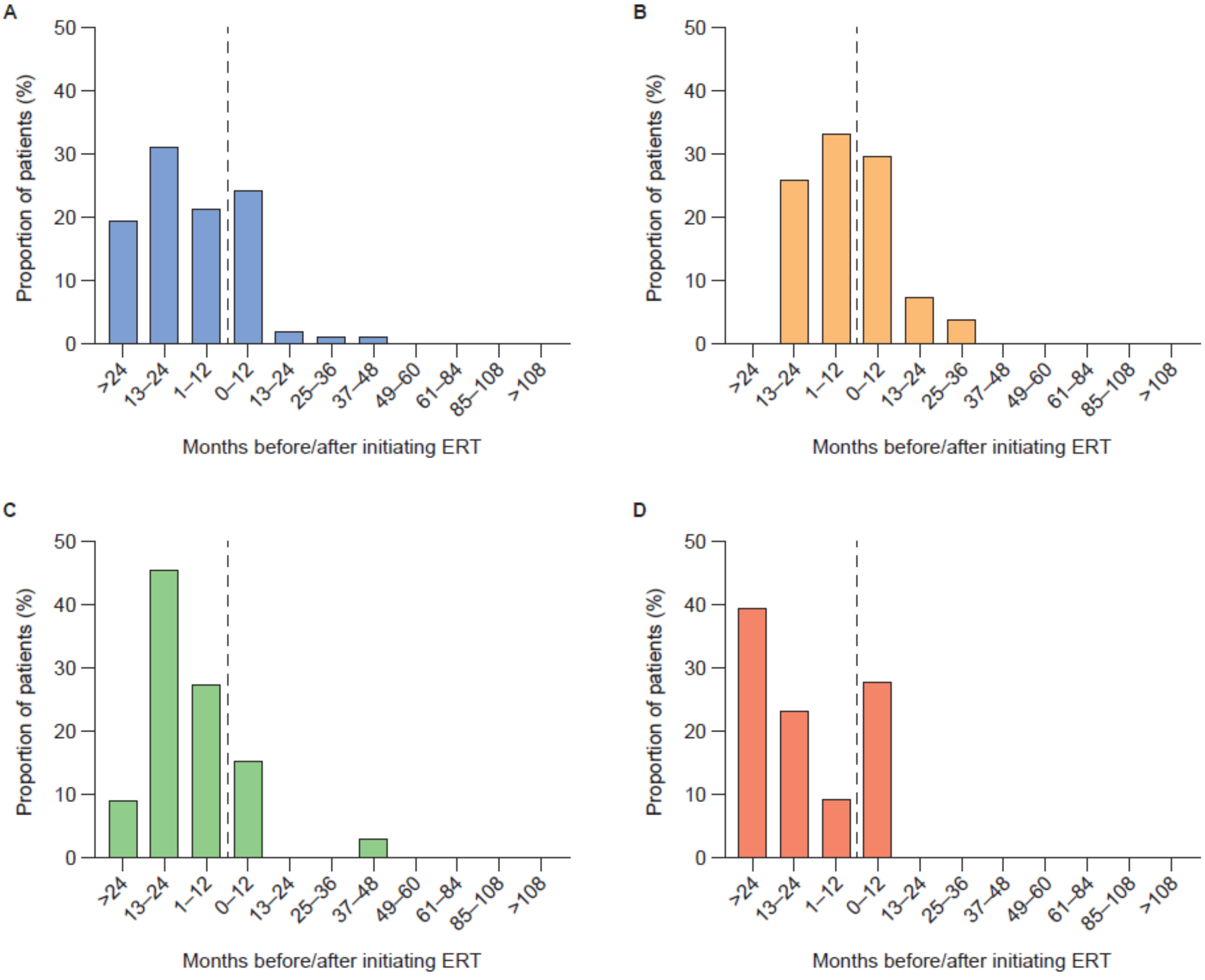

Results are presented for (**A**) all patients (n=103), (**B**) patients starting ERT aged <3 years (n=27), (**C**) patients starting ERT aged 3-6 years (n=33) and (**D**) patients starting ERT aged >6 years (n=43). No ENT symptoms were documented for 5 patients.

Proportion of patients who exhibited symptoms is shown. Dashed line indicates the point at which ERT was initiated.

Abbreviations: ENT, ear, nose, and throat; ERT, enzyme replacement therapy.

Figure S4. First Documentation of Infections Relative to Time of ERT Initiation

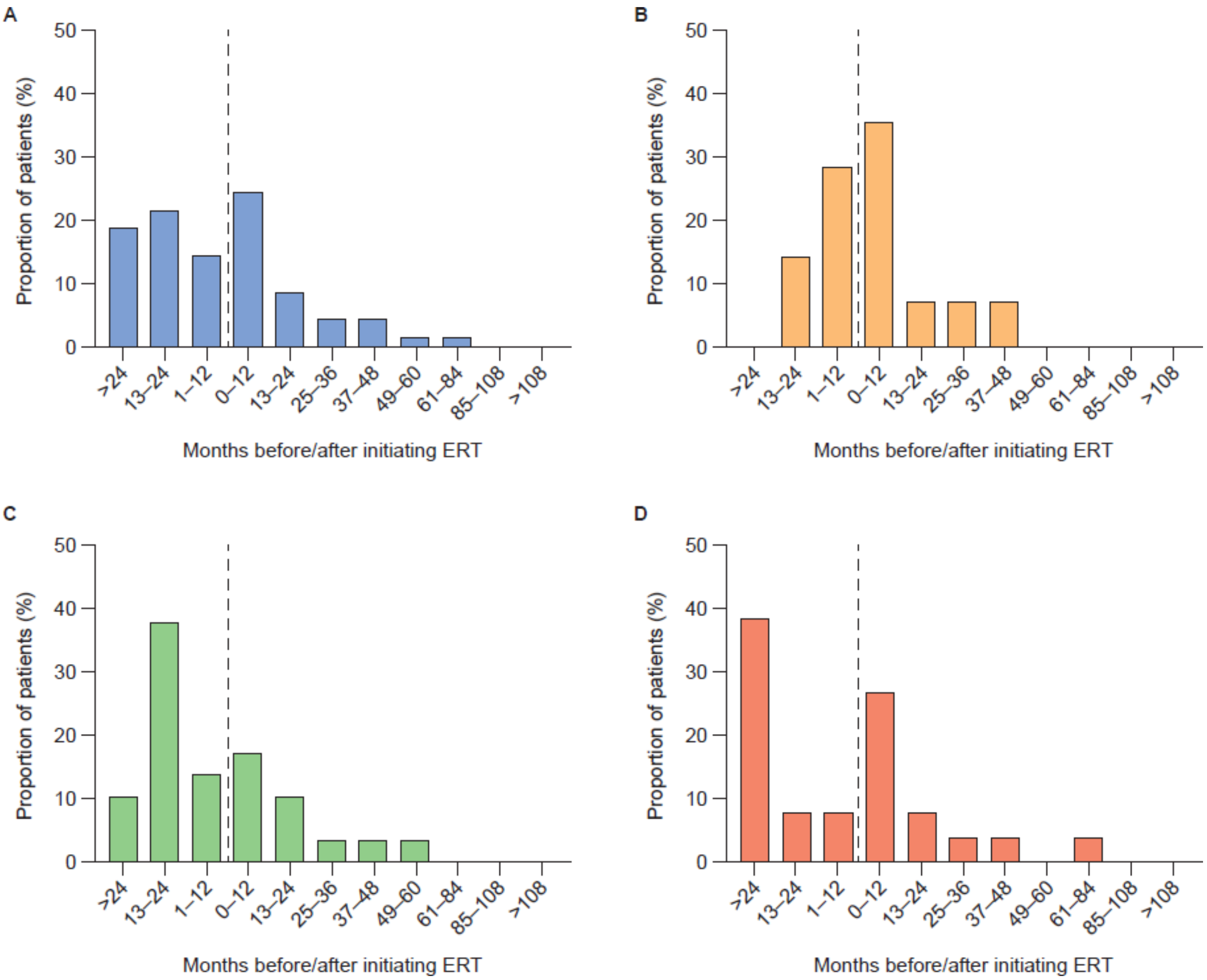

Results are presented for (**A**) all patients (n = 69), (**B**) patients starting ERT aged <3 years (n = 14), (**C**) patients starting ERT aged 3-6 years (n = 29), and (**D**) patients starting ERT aged >6 years (n = 26). No infections were documented for 39 patients.

Proportion of patients who exhibited symptoms is shown. Dashed line indicates the point at which ERT was initiated.

Abbreviation: ERT, enzyme replacement therapy.

Figure S5. First Documentation of CI Relative to Time of ERT Initiation

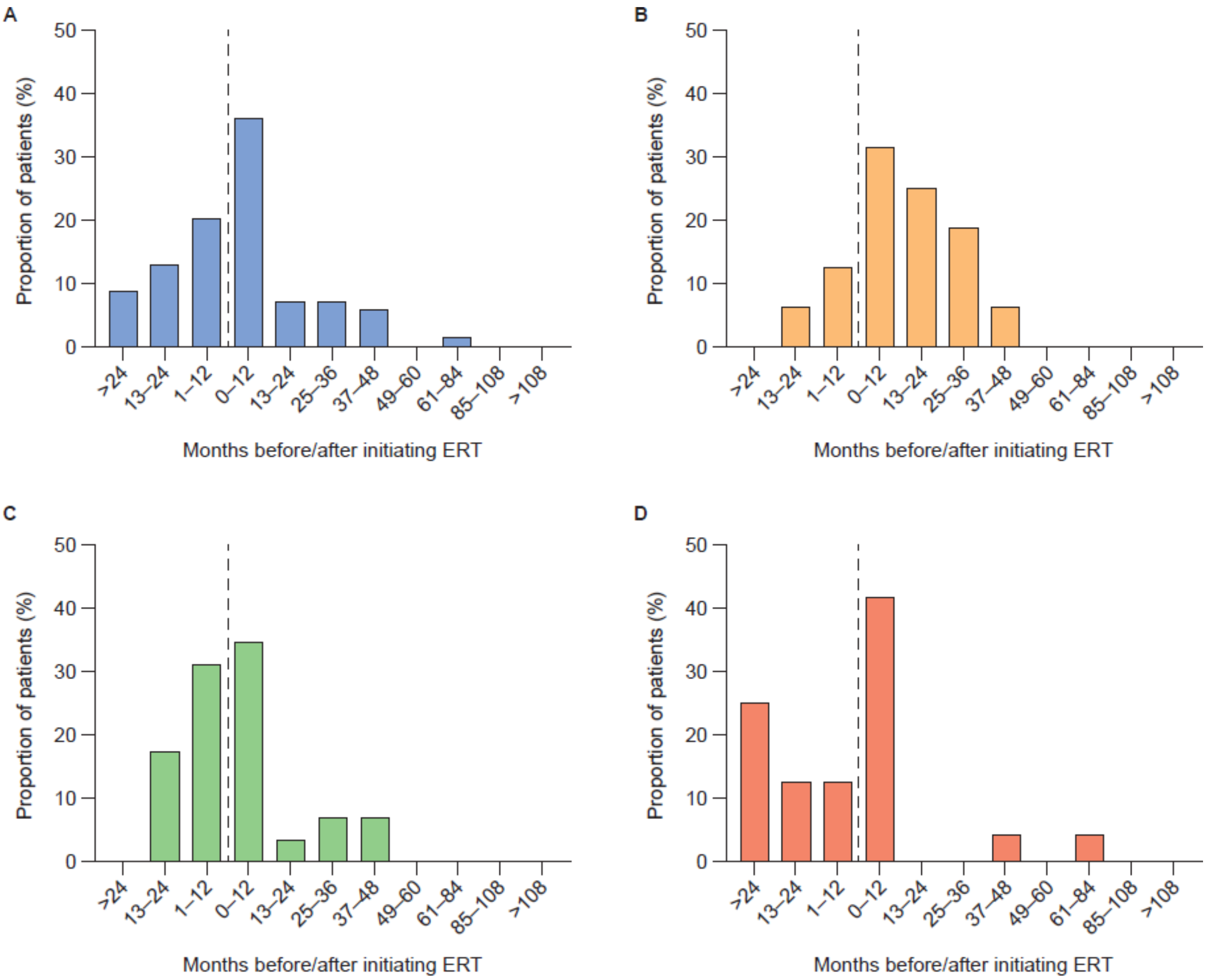

Results are presented for (**A**) all patients (n = 69), (**B**) patients starting ERT aged <3 years (n = 16), (**C**) patients starting ERT aged 3-6 years (n = 29), and (**D**) patients starting ERT aged >6 years (n = 24). No CI symptoms were documented for 39 patients.

Proportion of patients who exhibited symptoms is shown. Dashed line indicates the point at which ERT was initiated.

Abbreviations: CI, cognitive impairment; ERT, enzyme replacement therapy.
